# Feasibility of the Enhancing Participation In the Community by improving Wheelchair Skills (EPIC Wheels) program: study protocol for a randomized controlled trial

**DOI:** 10.1186/1745-6215-14-350

**Published:** 2013-10-24

**Authors:** Edward M Giesbrecht, William C Miller, Janice J Eng, Ian M Mitchell, Roberta L Woodgate, Charles H Goldsmith

**Affiliations:** 1 Department of Occupational Science and Occupational Therapy, University of British Columbia, Vancouver, Canada; 2 Department of Physical Therapy, University of British Columbia, Vancouver, Canada; 3 Department of Computer Science, University of British Columbia, Vancouver, Canada; 4 Faculty of Nursing, University of Manitoba, Winnipeg, Canada; 5 Faculty of Health Sciences, Simon Fraser University, Burnaby, Canada

**Keywords:** Cognitive computer games, Home-based training, Manual wheelchair, Older adults, Rehabilitation

## Abstract

**Background:**

Many older adults rely on a manual wheelchair for mobility but typically receive little, if any, training on how to use their wheelchair effectively and independently. Standardized skill training is an effective intervention, but limited access to clinician trainers is a substantive barrier. *Enhancing Participation in the Community by Improving Wheelchair Skills* (EPIC Wheels) is a 1-month monitored home training program for improving mobility skills in older novice manual wheelchair users, integrating principles from andragogy and social cognitive theory. The purpose of this study is to determine whether feasibility indicators and primary clinical outcome measures of the EPIC Wheels program are sufficiently robust to justify conducting a subsequent multi-site randomized controlled trial.

**Methods:**

A 2 × 2 factorial randomized controlled trial at two sites will compare improvement in wheelchair mobility skills between an EPIC Wheels treatment group and a computer-game control group, with additional wheelchair use introduced as a second factor. A total of 40 community-dwelling manual wheelchair users at least 55 years old and living in two Canadian metropolitan cities (*n* = 20 × 2) will be recruited. Feasibility indicators related to study process, resources, management, and treatment issues will be collected during data collection and at the end of the study period, and evaluated against proposed criteria. Clinical outcome measures will be collected at baseline (pre-randomization) and post-intervention. The primary clinical outcome measure is wheelchair skill capacity, as determined by the Wheelchair Skills Test, version 4.1. Secondary clinical outcome measures include wheelchair skill safety, satisfaction with performance, wheelchair confidence, life-space mobility, divided-attention, and health-related quality of life.

**Discussion:**

The EPIC Wheels training program offers several innovative features. The convenient, portable, economical, and adaptable tablet-based, home program model for wheelchair skills training has great potential for clinical uptake and opportunity for future enhancements. Theory-driven design can foster learning and adherence for older adults. Establishing the feasibility of the study protocol and estimating effect size for the primary clinical outcome measure will be used to develop a multi-site randomized controlled trial to test the guiding hypotheses.

**Trial registration:**

Clinical Trials NCT01740635.

## Background

Canada has a rapidly growing aging population; over the next 50 years, the proportion of older people is expected to double to more than one in four [1]. With age, the risk of a disabling health condition increases; mobility is the most prevalent area of impairment among older adults in Canada [2]. The number of wheelchairs provided to address mobility issues among older adults is also rising. In 2001, an estimated 81,000 Canadians 65 years and older required a wheelchair for mobility [3] – a rate four times the national average. A 2004 study [4] reported that among multiple assistive device users, the manual wheelchair (MWC) was considered third most important, behind eyeglasses and canes. However, the wheelchair also represents a substantial cost to consumers and the health care system. Beyond the cost of purchase, which varies from several hundred to thousands of dollars, our clinical experience has demonstrated that the process of assessment, procurement, fitting, and delivery can reach $10,000 or more. These systemic costs are squandered if older adults are unable to use their wheelchair effectively to participate in important activities of life.

Merely acquiring a wheelchair does not guarantee independence or satisfactory performance with functional activities. In particular, environmental factors, such as carpet, ramps, curbs, gravel, and poor sidewalk conditions, present barriers to mobility and self-propulsion. In Canada, over 90% of older MWC users experienced restricted performance in at least one major life activity [2] (compared with 15% of those without a mobility device) and nearly 60% require assistance from a family member or other care provider for even basic mobility [3]. Restricted mobility is associated with reduced participation and a loss in social connectedness [5], which can lead to feelings of isolation, stress, and low self-esteem, impacting overall quality of life [6]. A 2006 study of stroke survivors adjusting to wheelchair use identified substantial restriction in caregivers’ social roles and an increased burden of care [7]. In Canada, 25% of caregivers of the elderly are over 65 years themselves [1], and risk both acute and overuse injury when assisting with wheelchair use [8].

In addition, MWC users are at risk of tips and falls, which often result in injury. In Canada, the yearly incidence of tips or falls is estimated to be 5.2%, with 4.2% resulting in injury and 2.5% requiring a visit to an emergency department [9]. In the United States in 2005, wheelchair-related accidents resulted in one death per week and treatment for a hospitalized injury was estimated at $25,000 to 75,000 [10].

Providing comprehensive skills training is effective, but relies on considerable 1:1 training time. The Wheelchair Skills Training Program [11] is the only structured training program reported in the literature. An expert clinician provides personal training, typically requiring four to eight sessions of an hour or more. Several studies have demonstrated the Wheelchair Skills Training Program to be safe and practical [12,13], and randomized controlled trials report a significant improvement in skill capacity among adult MWC users during inpatient rehabilitation [14] and in the community [15]. Improvements in safety with skill performance have also been reported [16]. While the evidence indicates such training is effective, there has not been widespread clinical adoption and as a result older adults receive little or no structured wheelchair skills training. Even among younger populations receiving inpatient rehabilitation, only 17 to 18% of wheelchair users receive any formal training [17,18], which typically focuses on such skills as transferring from the wheelchair to the bed, toilet, or bathtub. A survey of older veterans who were prescribed a wheelchair post-stroke found 53% had received no instruction at all on wheelchair use [19]. Another study of US veterans reported that more than 50% had difficulty with even basic wheelchair propulsion, despite having access to a trained clinician and a custom-fitted wheelchair [20].

Several factors contribute to the current situation of inadequate training. First, many clinicians do not have sufficient knowledge of (or ability to demonstrate) wheelchair skills [12]. Second, other competing demands are often prioritized over wheelchair training during in-hospital rehabilitation. Older adults are frequently discharged with a temporary (standard) wheelchair and delivery of a customized wheelchair occurs in community settings, when therapists’ time is limited. Finally, funding for home care and community-based services in Canada and the United States has been in decline and is insufficient to support clinician-intensive training either before or after discharge [21,22]. Often, the time and travel demands for both consumers and clinicians make traditional skills training cost-prohibitive [23]. Long wait lists and the inaccessibility of rehabilitation services, particularly in rural areas, further exacerbate the problem [23,24].

Delivering rehabilitation training as a monitored or self-managed home program among older adults has been effective for a variety of outcomes including strengthening [25], physical activity [26], self-care [21,27] and exercise [28,29]. Home programs are advantageous because they allow privacy for the user, occur in a familiar context, can be conveniently integrated into the user’s schedule, and do not require the time, effort, and expense of travel [26]. Adherence to any intervention is critical to its effectiveness. A 2010 Cochrane review of exercise interventions found those interventions that incorporated social cognitive theory (that is, self-efficacy), were monitored, and increasingly graded complexity of the activity were more effective in improving adherence, frequency, and duration of exercise [30].

Computer-related devices are becoming increasingly useful for rehabilitation interventions, with advances in affordability, size, portability, accessibility, and user-interface simplicity. Computer and popular gaming systems have shown promising results in rehabilitation by casting therapy in a more engaging and enjoyable context. Their use for physical activity training in rehabilitation among older adults has demonstrated high participation rates, increased motivation, and tolerance for activity, and trends toward improvement in fitness [31].

The Enhancing Participation in the Community by Improving Wheelchair Skills program (EPIC Wheels) is an individualized home training program that optimizes learning for older adults while limiting the time demands of expert trainers. The program content and delivery was developed using principles of andragogy (adult learning) [32,33] and social cognitive (self-efficacy) [34,35] theory. Using an affordable computer tablet device, EPIC Wheels provides a customized and mobile structured training program for in-chair or tabletop use at home and in the community. A touch-screen audiovisual display features interactive training and practice activities, and wireless Internet connectivity enables trainee-trainer communication and remote trainer monitoring. The EPIC Wheels program is 1 month long and includes a minimum of 10 hours of training and practice. Training includes two personalized sessions with a wheelchair expert; one at the outset and the second at the mid-point of the program.

Prior to moving to a large-scale randomized controlled trial (RCT), it is not only critical but also prudent to ensure that the feasibility indicators and the proposed clinical measures are sufficiently robust. This paper describes the objectives and design of the EPIC Wheels feasibility study that will be used to construct and implement a comprehensive, multi-site RCT trial that directly assesses the guiding hypotheses.

### Study objectives

#### Feasibility indicators

These have been selected to assess the feasibility of study methods and procedures, including:

• Process issues of subject recruitment, consent, retention, and perceived benefit.

• Resource issues of treatment adherence, burden of data collection, incorporating a health utility index, and intervention burden.

• Management issues of tablet reliability, subject processing, and protocol administration.

• Treatment issues of safety, response, and treatment effect.

#### Primary clinical outcome

The aim of the study is to evaluate the effect of EPIC Wheels on wheelchair skill capacity and obtain an estimate of the treatment effect size.

#### Secondary clinical outcome

The secondary aims are to evaluate the effect of EPIC Wheels on wheelchair skill safety, wheelchair use confidence, satisfaction with activity performance, mobility, divided-attention, and health-related quality of life.

## Methods/design

### Trial design

**Figure 1:**
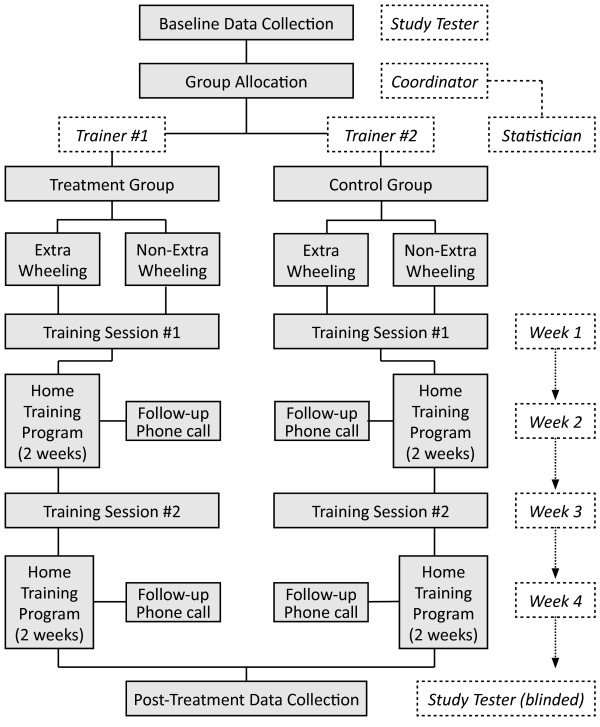
Trial procedure.

This study uses a two-site RCT to compare differences in older adults’ wheelchair mobility skills between an EPIC Wheels (treatment) group and a cognitive training (control) group, introducing 'extra wheeling’ as a second factor. A 2 × 2 factorial design randomly assigns subjects using a 1:1:1:1 allocation ratio between four groups: EPIC Wheels, EPIC Wheels with extra wheeling, cognitive training, and cognitive training with extra wheeling. To support balance between groups and masking of assignment, a central computerized randomization algorithm was designed by our statistician, with an undisclosed block size and stratified by site (*n* = 20 at each site). Once subjects are enrolled, a tester collects baseline data before a study coordinator contacts the statistician to obtain group assignment. A trainer will initiate contact with the subject and implement the 1-month program, after which time the tester will re-administer the outcome measures (see Figure 1). To address bias, subjects will be instructed not to discuss their program; separate trainers will be used for the treatment and control groups at each site; and testers will be blinded to group allocation. A protocol is included for control group trainers to identify the potential value of the control intervention for wheelchair mobility and strategies to prevent participant attrition.

### Ethics

The protocol for this study has been approved by the Research Ethics Boards at the University of British Columbia (Approval number, H12-02043) and the University of Manitoba (Approval number, HS15818), as well as the research review committee of the regional health authority for each site. All study subjects, and their caregivers, will provide informed consent prior to enrolment. The study was funded through a peer-reviewed operating grant from the Canadian Institutes of Health Research (MOP-123240).

### Participants

A total of 40 community-dwelling MWC users living in two Canadian cities (20 at each site) will be recruited on a voluntary basis. Each site will have 20 participants, with five participants in each of four groups: treatment with extra wheeling, treatment without extra wheeling, control with extra wheeling, and control without extra wheeling. To optimize the impact of the treatment, individuals with less than one year of MWC use will be recruited. Novice users are still developing routines and patterns of wheelchair use and are potentially more amenable to adapting their mobility techniques [12]. Subjects will be ≥55 years old; live in the community within the metropolitan boundaries; self-propel a MWC ≥1 hour/day; use their MWC for mobility inside and outside their home; have used a MWC for <1 year; and have a caregiver willing to attend training sessions and supervise home training. There are no specific diagnostic criteria for enrolment; however, subjects must be able to propel a wheelchair with both hands. Individuals will be excluded if they cannot communicate and complete study questionnaires in English; anticipate a health condition or procedure that contraindicates training (for example, surgery scheduled which would impair physical activity); or are currently receiving outpatient therapy that includes wheelchair mobility training. A caregiver for each participant will also provide consent to participate in the study, and both subject and caregiver will indicate whether they are willing to be contacted for follow-up after the study is complete.

### Sample size

To address the feasibility indicators, the number of subjects is large enough to represent the target population and assess the feasibility criteria [36]. In addition, the sample size is powered to detect a statistically significant difference between groups and provide a reasonable estimate of a treatment effect. With a 2 × 2 factorial design, sample size can be calculating using each main factor independently, and then determined by selecting the larger of the two estimates [37]. Our calculations are based on the primary clinical outcome measure (the Wheelchair Skills Test version 4.1, WST), as this will be used in subsequent EPIC Wheels studies. Research in the field of wheelchair skills training is still maturing, with three published studies using actual users, and given the novel home program approach an α of 0.10 was selected to ensure a potentially beneficial treatment effect would not go undetected (Type I error rate). Given the absence of data regarding the impact of extra wheeling, we opted to use a comparable effect size (*f* = 0.54) in the second factor calculation. To minimize the risk of identifying such an effect merely by chance (Type II error), the study is powered at 90%. Based on a sample size calculation for analysis of covariance (ANCOVA) each of the four groups would require eight subjects for a total of 32 participants. In previous Canadian trials, a 9 to 18% dropout rate has been reported; conservatively adjusting for a 25% dropout rate (32/0.75), a total study *N* of 44 is planned (*n* = 11 for each group).

Previously published studies have used younger or mixed age populations; data specific to older adults are not available. We obtained permission to use a data subset (adults >50 years) from a yet-to-be published study (F Routhier, personal communication) that provided WST change scores following training and powered our study to capture a comparable change (*m* = 9.3%; *s* = 9.5%). A difference of 9.3% corresponds to an acquisition of three additional skills on the WST; previous studies report that subjects perceive a clinically important difference with such improvements [17,18]. In fact, the skills performed on the WST are sequenced from simple (for example, rolling forwards) to moderate (for example, propelling on carpet) to complex (for example, ascending a 10° ramp). Research literature reporting on MWC use among older adults specifically implicates carpet, inclines, curbs, gravel, and poor sidewalk conditions as barriers to independent mobility [7]. Acquisition of even one of these important skills could quite reasonably represent a minimally clinically important difference (MCID) to wheelchair users, their caregivers, and those providing training. No formal MCID has been established for the WST; however, using data from a Canadian trial [18], a smallest detectable difference calculation suggests that a difference of 9.2% would exceed any measurement error or noise [38]. A reliability change index calculation indicates that 3.0% is the minimal detectable change required and is a reasonable proxy for MCID [38].

### Procedure

Subjects attend an initial session with their respective trainer for 90 to 120 minutes, including orientation to the computer tablet. Subjects are provided with a prepared study tablet and printed reference handbook to engage in their home training program for 2 weeks, with a minimum of 150 minutes of tablet-based activity per week. After 2 weeks, subjects attend a second session with their trainer for 60 to 90 minutes and then continue with their home training program for an additional 2 weeks. The trainer makes follow-up telephone contact at the end of weeks 1 and 3.

### Extra wheeling

The EPIC Wheels program requires trainees to engage in wheelchair activity that they might not otherwise undertake. While it is unlikely that such additional wheelchair use alone would substantially increase skill acquisition or proficiency, to address this potentially confounding variable subjects are randomly allocated into extra wheeling and non-extra wheeling subgroups. Subjects in the extra wheeling subgroups are asked to perform an additional 75 minutes of unstructured wheelchair wheeling per week, which can occur in their home or in the community. Extra wheeling is defined as participants propelling their wheelchair in addition to what they would typically do in the course of daily activities. For these subjects, the training tablet presents a prompting question every 24 hours asking how many minutes of extra wheeling they have performed. Subjects toggle up and down in 5-minute increments and enter their data before the tablet returns to the training activities. Trainers can encourage subjects to increase their extra wheeling activity during follow-up phone calls and training visits if target levels have not been met.

### Intervention

#### Treatment group (EPIC wheels)

EPIC Wheels trainers are occupational therapists with ≥5 years of clinical experience in wheelchair provision and training, who have received a 2-day comprehensive orientation program. Each training session is administered using a protocol and checklist. Trainers select a subset of skills and training activities to incorporate into the home training program based on ability, safety, and relevance for the subject. The EPIC Wheels home program includes a comprehensive, structured library of educational material and training activities, organized in a hierarchy from simple to complex. Trainers monitor a subject’s progress through online access to tablet usage data and re-assessment during the second training session, revising the home program with more advanced skills and activities.

The home program component is delivered using a 10-inch (≈25 cm) portable ASUS® computer tablet (ASUSTek, Markham, ON). The tablet is menu driven and interactive, using a touch-screen interface. Training is provided in a multi-modal format with illustrations and videos, allowing detailed step-by-step guidance and slow-motion demonstrations. Female and male actors, both close to 70 years of age, were used to provide age-appropriate models in the videos. Practice activities can be clearly demonstrated and include imitative, function-based, and interactive game-related activities. The tablet is mounted on a rigid platform with a simple strap that wraps around the subject’s thighs for use in a wheelchair. A 'progress’ icon provides daily updates on the number of minutes practiced per week to reinforce adherence. Subjects can exchange voicemail with their trainer using an integrated applet. Subjects receive a mobile Internet device for voicemail and data transfer or update capability, but the tablet operates as a single-function device with all other applications disabled.

#### Control group (cognitive training)

Control group subjects also attend two sessions with their trainer to address attention balance through exposure to study personnel. In addition, they receive an identical tablet pre-loaded with computer games to account for tablet device and activity exposure. Nine different games address problem solving (for example, Tetris, Cogs); word, math, and memory challenges (for example, Scrabble, Sudoku); and dexterity or response skills (for example, Marble Saga, Cut the Rope). Each training session is administered using a protocol and checklist. During the first session, the trainer discusses the potential benefits of computer-game training on cognition and motor function, and how these can positively impact wheelchair mobility. Trainers provide an orientation to the cognitive games and operation of the tablet device. During the second session, the trainer discusses subjects’ current community activities and experience using the wheelchair via a structured discussion guide, and provide verbal information related to barriers encountered, as well as any additional review or training related to the cognitive training games. To minimize attrition and provide wheelchair skill-specific information, the consent form informs control group subjects that they will receive a DVD with a condensed EPIC Wheels education program after the post-intervention data collection is complete.

### Training schedule

The home training schedule for both groups targets a minimum of 1 or 2 sessions/day, 15 to 30 minutes in length, at least 5 days per week (minimum 150 minutes/week). These guidelines are based on the National Blueprint consensus document on promoting physical activity for adults over 50 years, which advocates that lifestyle- or endurance-related activity of moderate intensity should be undertaken for at least 30 minutes (in bouts of at least 10 minutes) 5 to 7 days per week [39]. The minimum training time is at least comparable to 1:1 training time in other clinical studies using structured wheelchair skills training, where a significant improvement in skill capacity was observed [17-19]. Adherence to this schedule is achievable, as demonstrated by a study of home-based training program for improving hand function among stroke survivors (*n* = 77; *m* = 57 years) which obtained 96% compliance for 1.3 hours of training per day, 7 days per week over 5 weeks [40].

### Safety

The EPIC Wheels program incorporates extensive safety-related material, including teaching the safest mobility strategies; use of safety equipment; recognizing unsafe situations; and seeking assistance when skills are insufficient to address environmental barriers. At the initial training session, subjects are provided with protective wheeling gloves. Caregivers attend and participate in both training sessions and encouraged to supervise home training activities. A fitted spotter’s strap and demonstration and instruction are provided for caregiver use during training to prevent wheelchair tips. Operating a wheelchair in the community carries innate risks that cannot be entirely eliminated; however, EPIC Wheels offers education and training designed to minimize risks of wheelchair operation. This knowledge should reduce the risk of a fall or injury that subjects might be exposed to in their everyday use of a MWC had they not received this program. Any unsafe performance observed during training is addressed immediately with corrective feedback. Subjects are encouraged to contact the coordinator immediately if they experience unusual discomfort, pain, or physical symptoms. A data and safety monitoring board, consisting of a statistician, a physiatrist, and a physical therapist, review accumulating indicator data and advise the investigators regarding safety issues, evidence of benefit, and need for modification to the study design [41]. Adverse events are documented by the trainer and reported to the data and safety monitoring board and the ethics review board.

### Computer technology uptake

The use of a computer tablet to deliver the home training program offers advantages of portability, audiovisual versatility, flexible configuration, and real-time updating. Although older adults are less likely than younger people to embrace technologies, such as computers, their use of computers continues to grow. Studies in the United States between 2006 and 2010 found 84% of those over 60 years had experience with computers [42] and 40% of those over 65 years are regular computer and Internet users [43]. Use of a tablet involves some new learning, and age-related declines in memory and fluid intelligence may restrict uptake. These issues are addressed through self-paced training, structures for successful experiences to build confidence, and by adapting the interface for familiarity and ease of use with minimal memory requirements [44,45]. Consumer, caregiver, and clinician input were incorporated during the EPIC Wheels program development to ensure the delivery format addresses these concerns.

### Data collection

At baseline, wheelchair device characteristics are collected using a modified wheelchair specification form [46]. Descriptive characteristics including age, sex, marital status, highest level of education, primary diagnosis related to MWC use, length of time using the MWC, and propulsion method are collected along with cognitive status measured using the Standardized Mini-Mental Status Exam [47]. Handgrip strength has been demonstrated to be an accurate surrogate measure of overall strength [48] and is measured using a Jamar^TM^ 5030J1 dynamometer (Sammons Preston Rolyan, Chicago, IL).

Feasibility indicators for process, resources, management, and treatment characteristics are collected during study administration and at the study’s end. Process indicators include recruitment, consent, and retention rates at each site. Retention is a critical factor for subsequent clinical trials; follow-up with all discontinued participants regarding their reason for dropout will inform future study design and sample size calculations. A post-treatment participant questionnaire is used to evaluate perceived benefit. Resource indicators are monitored by tracking study staff time logs and tablet usage data. Management issues are evaluated using a trainer evaluation form and by tablet reliability or loss data. Treatment indicators include reporting of adverse events and statistical analysis of the treatment effect.

The clinical outcome measures are collected at baseline (pre-randomization) and post-treatment (1 month). The primary outcome is wheelchair skill capacity, measured using the Wheelchair Skills Test 4.1 (WST) [14]. The WST is a structured assessment with 32 discrete mobility skills required to perform social roles in the community, each scored dichotomously as pass or fail, producing a total skill capacity score (0 to 100%) with a higher score reflecting more skills acquired. The WST is sufficiently sensitive to detect proximal effects of training; can be completed in approximately 30 minutes; and does not demonstrate floor or ceiling effects [16]. Two systematic reviews of available wheelchair skill outcome measures confirmed that the WST has the strongest psychometric properties and has been used most extensively in clinical trials [49,50]. The WST has demonstrated reliability for test-retest (intraclass correlation coefficient (ICC) = 0.90), intra-rater (ICC = 0.96), and inter-rater (ICC = 0.97) administration [16]. Construct validity has been supported by significant relationships with predictive variables of age, sex, MWC experience, diagnosis, and use of a lightweight wheelchair, which together accounted for 35% of variability in WST score using multiple regression (adjusted *R*^2^ = 0.35) [16]. Concurrent validity has been established through positive correlation with two criterion measures: therapists’ global assessment of user ability (*R*_S_ = 0.39 - 0.40) and the Functional Independence Measure (admission score *R*_S_ = 0.38; discharge score *R*_S_ = 0.31) [16,51].

Secondary clinical outcomes reflect more distal impacts of the intervention. Given the dearth of evidence in the literature, there is substantial value in understanding the relationship between skill acquisition and safety, confidence, community participation, mobility, and utility. Seven secondary measures contribute to discerning a clinically important impact of the EPIC Wheels intervention.

• WST 4.1 skill safety. The WST also provides a total skill safety score (0-100%) reflecting the number of skills addressed in a safe manner (higher score indicates greater safety), regardless of whether the skill is acquired or not. This is of considerable importance, since training also involves learning to recognize risks and limitations.

• Wheelchair Outcome Measure (WhOM). The rehabilitation literature strongly suggests the use of measures of user-identified activities of relevance and perceived satisfaction with performance [52-54]. Using an interview format, MWC users are asked to identify relevant activities and rate them for both importance and satisfaction, using an 11-point scale (0 to 10), with higher scores indicating greater importance or satisfaction. The WhOM demonstrates reliability and validity in use among older adults (Test-retest ICC = 0.77 to 1.00; correlation with QUEST [55]*r*_*s*_ = 0.36 to 0.45) [56].

• Wheelchair Use Confidence Scale for Manual Wheelchair Users (WheelCon-M 3.0). Self-efficacy has been identified as a key component in the performance of wheelchair mobility skills [57] and preliminary research has suggested that standardized training can increase wheelchair confidence among older adults [58]. The WheelCon is a self-report questionnaire composed of 65 statements related to confidence using a wheelchair in activities and environments, each rated on a scale from 0 (not confident) to 100 (completely confident), producing a total mean score of 0 to 100 [59]. A 2010 study evaluated test-retest reliability (ICC = 0.84) and significant correlation with comparison measures supporting its validity [60].

• Life-Space Assessment (LSA). The LSA is a 20-item questionnaire that tracks the wheelchair user’s travel on a continuum of five environments from the home to outside of town [61], and capturing mobility habits over a 4-week period, including frequency of travel and level of assistance required. Scores vary from 0 to 120 and are weighted for frequency and level of assistance, with higher scores reflecting further distance from home, greater frequency of travel, and less assistance required. Evaluation of the LSA among power wheelchair users found excellent test-retest reliability (ICC = 0.87) [62].

• Health Utility Index Mark 3 (HUI3). Health utility measurement is useful in performing cost-utility and cost-effectiveness analyses of new rehabilitation interventions. National guidelines for healthcare economic analyses strongly advocate the use of a validated measure of health-related quality of life, which can be converted to quality-adjusted life years gained, so as to fully inform funding decisions [63]. The HUI3 is a brief questionnaire that asks subjects about their health status, deriving both single- and multiple-attribute utilities to ascertain quality-adjusted life years [64], and meets the criteria for a valid health-related quality-of-life utility score [65]*.* Each single-attribute utility is scored between 0.00 and 1.00 and the multiple-attribute utility scale is scored from -0.36 to 1.00, with higher scores reflected better health and quality of life. Our study is not sufficiently powered to undertake a cost-utility analysis, but will determine the feasibility of collecting cost-utility data in a larger RCT and estimate what changes in health-related quality of life might be anticipated.

• Wheeling While Talking test (WheelTalk). Wheelchair mobility is a complex skill and prone to risk of tips, falls, and potential injury, particularly with older adult users, where motor and cognitive function may decline with age. The WheelTalk is a new divided-attention assessment for wheelchair users. Scoring reflects the additional number of seconds required to complete a slalom course during the dual-task versus motor task-only condition, with a larger time differential indicating poorer performance. Initial evaluation with residents in a long-term care facility demonstrated reliability for test-retest (ICC = 0.92), intra-rater (ICC = 1.00) and inter-rater (ICC = 1.00) administration [66]. Collecting data in this study will allow investigation of associations between performance and wheelchair skill, safety, and other demographic factors, contributing to validation of this tool.

• Data logger. To capture level of activity while in the wheelchair, a data logger (three-axis self-recording accelerometer X16-1C, Gulf Coast Data Concepts, Waveland, MS) is mounted on the drive wheel spokes of each subject’s wheelchair. The data logger is a small, unobtrusive 'black box’ that collects drive wheel movement via an accelerometer over a period of 6 to 10 days. Distance travelled and bouts of travel will be calculated, with larger values reflecting more activity. Collecting these data will allow description and comparison of wheeling patterns and extent of activity among participants. Data loggers are installed at the first training session and removed at the second training session, approximately 2 weeks later.

### Statistical analyses

Study analyses will consider study feasibility indicators as well as clinical (statistical) outcomes. Means and standard deviations (for continuous variables) and frequencies and proportions (for categorical variables) will be used to summarize case mix and outcome variables for groups. Qualitative comparison of variable balance will be made, including determination of prognostic importance [36]. Descriptive statistics of training time and number of training sessions (collected via tablet usage data) will be used to evaluate adherence and explore potential associations between training intensity and measured outcomes.

A detailed description of the feasibility indicators and measurement criteria is provided in Additional file 1. Feasibility indicators will be treated as binary, with 'success’ indicating that the protocol is sufficiently robust to move forwards with the large RCT with only small or no adaptation required, and 'revise’ indicating a need for more substantive change before proceeding. The number and extent of objectives requiring revision will determine whether the feasibility study data can be conflated with those produced in a larger subsequent RCT.

Post-treatment WST skill capacity scores will be compared in the treatment and control groups using analysis of covariance (ANCOVA), controlling for baseline score as a covariate for the primary analysis [67]. Borm *et al*. [68] demonstrate that correlation between pre- and post-intervention scores (*ρ*) influences analysis power; when *ρ* > 0.5, change score is more powerful than direct post-intervention comparison. However, when *ρ* lies between 0.2 and 0.8, ANCOVA (controlling for baseline score) further reduces the required sample size by 10 to 40% over change score. Given that preliminary data suggest *ρ* ~ 0.5 (F Routhier, personal communication), ANCOVA should provide the most powerful analysis, in addition to reducing error variance and allowing modification when statistical assumptions are not met [69]. Unequal cell sizes will be accommodated using Method 1 adjustment [70] and diagnostic assessments made for model assumptions. Significance testing (*P*) and marginal means with 95% confidence intervals will be estimated. Effect size (partial *η*^2^) will be calculated as a ratio of the effect and total sums of squares, with a 95% confidence interval. To preserve prognostic balance, primary analysis will be based on intention to treat; however, since one objective is to estimate the treatment effect, secondary analysis on a per-protocol basis (subjects who adhere to treatment) will also be conducted, for comparison [71].

One argument for potential improvement in wheelchair skills using the EPIC Wheels program is the inducement of subjects to use their wheelchair more, rather than the specific content of the training program. Our factorial design, with extra wheeling as a second factor, will attempt to address this question. Initial analysis will contrast the two main effects: training (EPIC Wheels versus cognitive games) and extra wheeling (yes versus no). Secondary analysis of the interaction contrast between factors may delineate whether additional wheeling has a synergistic or antagonistic effect on the training program.

ANCOVA will be used to compare post-treatment scores between groups for WST skill safety, wheelchair confidence (WheelCon), safety risk (WheelTalk), and mobility (LSA) as a secondary analysis. In each case, baseline score will be used as a covariate. Subjects’ satisfaction with performance of meaningful activities (WhOM score) will be analyzed using analysis of variance (ANOVA). Mean administration time for the HUI3 will be calculated and a preliminary evaluation of change in the treatment group will be conducted. Endpoints and change analyses will also be conducted to compare their precisions with ANCOVA. Summary and descriptive statistics for distance and speed (mean and standard deviation) and bouts of activity (counts) will be calculated for the data logger data.

## Discussion

The EPIC Wheels study is intended to establish the feasibility of a novel intervention designed to improve wheelchair skills and safety among older adult manual wheelchair users. The EPIC Wheels program is innovative in several respects. First, it offers evidence-based skills training in a home training format that has greater potential for clinical uptake. While structured training has been demonstrated to improve skill capacity, access to and related costs of one-on-one training with an expert clinician has restricted widespread adoption. Consolidating the majority of training in a home program with remote electronic monitoring by the clinician makes for a more viable option from an economic and convenience standpoint. Second, EPIC Wheels has a theory-driven design to increase effectiveness among older adults and enhance program adherence, explicitly incorporating principles of social cognitive theory (self-efficacy) and andragogy (adult learning) into the content and delivery format. In addition, older adult stakeholder groups including MWC users, their caregivers, and clinician trainers, were intimately involved during the 1-year program development process. Research related to capacity and a principled teaching method for older adults to learn wheelchair mobility skills is sparse, and this study may provide important new evidence. Third, the use of commercially available and relatively inexpensive tablet technology provides a flexible and versatile mobile training interface. Trainees can practice with the tablet on their wheelchair in their home or community environment and communicate remotely with their trainer, while clinicians can monitor training activity and communication through a web-based interface at their convenience. Finally, the factorial study design will allow broader inferences about the contributions of increased wheelchair activity and the specific attributes of the EPIC Wheels training program, as well as an interaction between these factors.

Owing to the nature of feasibility studies, several inherent limitations arise and will be addressed in the larger multi-site RCT study to follow. The potential for recruitment of novice older adult MWC users is unknown: this is an important feasibility indicator to address for future planning. Assessment of skill retention will not be addressed at this stage. Control group subjects may be more likely to drop out. Providing control group trainers with structured strategies to maintain subject enthusiasm; weekly subject contact; engaging and fun cognitive computer games, and provision of a condensed training DVD at the study conclusion will help mitigate this risk. Demonstration of a treatment effect may lack generalizability, owing to the restricted age group being targeted. Age is inversely related to motor skill acquisition, including wheelchair mobility skills. We anticipate that a training program proven to be effective with older adults should have at least as large a treatment effect in younger and stronger individuals; however, this assumption is as yet untested.

The feasibility indicators of this study will be used to construct and implement a comprehensive, multi-site RCT trial that directly measures the guiding hypotheses. This subsequent study will address skill retention using a 6-month follow-up evaluation. A large RCT will directly address evaluation of important changes in wheelchair skill and safety; wheelchair use confidence; mobility and community participation; and cost-effectiveness. Evidence for the effectiveness of EPIC Wheels would inform clinical best practice and provide justification for a pilot project where a single trainer coordinates service to a large group of wheelchair users across a broad geographical area. EPIC Wheels has great potential for use across age and diagnostic groups, including training for individuals who live in rural and remote locations where access to rehabilitation programs is not practical. EPIC Wheels provides versatility, as it can be delivered on multiple platforms including computers, tablets, smart phones, and traditional DVD. Furthermore, the inherent capacity of the tablet device (that is, GPS, accelerometers) can be incorporated into future EPIC Wheels versions to collect real-time user data on wheelchair performance; to create interactive training activities or games; to evaluate skill performance; and integration with other commercially available technology.

## Trial status

Approval has been obtained from the university Research Ethics Boards and regional health authority Research Review Committees at both sites. All study staff have been hired and trained at both sites and recruitment is currently underway.

## Abbreviations

ANCOVA: Analysis of covariance; ANOVA: Analysis of variance; EPIC Wheels: Enhancing participation in the community by improving wheelchair skills; HUI3: Health utilities index mark 3; ICC: Intraclass coefficient; LSA: Life-space assessment; MCID: Minimally clinically important difference; MWC: Manual wheelchair; RCT: Randomized controlled trial; WheelTalk: Wheeling while talking test; WhOM: Wheelchair outcome measure; WST: Wheelchair skills test, version 4.1.

## Competing interests

The authors declare that they have no competing interests.

## Authors’ contributions

WCM was responsible for administration of the grant, oversight of the study, and first site lead. JJE provided guidance with the research design and implementation. IMM was instrumental in the development of the tablet software applications and provided ongoing technology design and support for the computer tablet software application. CHG provided expertise in the RCT design and analysis as well as cost-utility and cost-effectiveness evaluation. RLW contributed to the program evaluation and development research phase. EMG was primarily responsible for development of the intervention, pilot testing, and the second site lead. EMG wrote the first draft of the manuscript and all authors reviewed and contributed to the final version.

## Supplementary Material

Additional file 1 Detailed description of feasibility indicators and measurement criteria. Click here for file
