# Supplementary material for: Feasibility of the Enhancing Participation In the Community by improving Wheelchair Skills (EPIC Wheels) program: study protocol for a randomized controlled trial
Source: Trials. 2013 Oct 24;14:350. doi: 10.1186/1745-6215-14-350 (PMC3874600; doi:10.1186/1745-6215-14-350)
Supplement: Additional file 1 — Detailed description of feasibility indicators and measurement criteria. [file 1745-6215-14-350-S1.docx]

**Additional file**

**Detailed Description of Feasibility Indicators and Measurement Criteria**

| **Feasibility Component** | **Indicator** | **Criteria for Success** |
| --- | --- | --- |
| ***Process*** | | |
| Recruitment rate | # of subjects recruited | 3 subjects/month/site:  Total of 36 over 6 months |
| Consent rate | % of subjects consenting | < 10% subject refusal |
| Retention rate | % of subjects with T_2_ data collected | Complete T_2_ data collection with at > 80% of subjects |
| Perceived benefit | Post-treatment Participant Questionnaire  Qualitative Interviews | > 85% of responses will be  “strongly agree/agree”  Qualitative analysis will inform clinical importance |
| ***Resources*** | | |
| Treatment adherence  (Experimental Group)    (Control Group) | Attend both training sessions  Meet minimum practice time guidelines  Both social visits conducted | > 85% of subjects  > 85% of subjects  > 85% of subjects |
| Data collection: Subject & Tester burden | Data T_1_ collection  Data T_2_ collection | > 85% of subjects complete in ≤ 2 h  > 85% of subjects complete in ≤ 1.5 h |
| Collection of HUI data | Administration  HUI pre/post score | Mean HUI administration is < 10 minutes  Statistically significant change between T_1_ & T_2_ |
| Trainer burden | Time spent on monitoring intervention & subject | Mean time spent per subject is  < 2 hours |
| ***Management*** | | |
| Tablet reliability | Downtime due to technical or mechanical issues | > 90% of subjects are not without a tablet for > 2 days |
| Equipment loss/damage | Tablet is lost/unusable | < 2 tablets lost over study |
| Subject processing time | Time from initial contact to enrolment | Mean time is < 10 days at each site |
| Treatment administration issues | Post-treatment Evaluation Form (Study Trainer) | Any issues identified can be modified without substantial changes to the protocol |
| **Treatment** | | |
| Safety (Home program) | Adverse events during home training | No major injuries or adverse events reported |
| Safety (Data Collection & Training) | Adverse events during assessment or training | No major injuries or adverse events reported |
| Treatment response | ANCOVA comparison between groups | A significant difference between groups identified |
| Dose level response | Correlation between total training time and WST change score | Minimum practice time guidelines sufficient for a treatment effect |
| Treatment effect & variance | Estimate of effect size and variance for future sample size/power calculations | Data on all relevant variables |
